# Activation of the Renin-Angiotensin System Promotes Colitis Development

**DOI:** 10.1038/srep27552

**Published:** 2016-06-08

**Authors:** Yongyan Shi, Tianjing Liu, Lei He, Urszula Dougherty, Li Chen, Sarbani Adhikari, Lindsay Alpert, Guolin Zhou, Weicheng Liu, Jiaolong Wang, Dilip K. Deb, John Hart, Shu Q. Liu, John Kwon, Joel Pekow, David T. Rubin, Qun Zhao, Marc Bissonnette, Yan Chun Li

**Affiliations:** 1 Department of Pediatrics, Shengjing Hospital, China Medical University, Shenyang, Liaoning, 110004, China; 2 Department of Medicine, Division of Biological Sciences, The University of Chicago, Chicago, IL 60637, USA; 3 Department of Endocrinology, Shengjing Hospital, China Medical University, Shenyang, Liaoning, 110004, China; 4 Department of Pathology, Division of Biological Sciences, The University of Chicago, Chicago, IL 60637, USA; 5 Department of Cardiology, The First Affiliated Hospital, China Medical University, Shenyang, Liaoning, 110001, China; 6 Department of Biomedical Engineering, Northwestern University, Evanston, IL 60208, USA

## Abstract

The renin-angiotensin system (RAS) plays pathogenic roles in renal and cardiovascular disorders, but whether it is involved in colitis is unclear. Here we show that RenTgMK mice that overexpress active renin from the liver developed more severe colitis than wild-type controls. More than 50% RenTgMK mice died whereas all wild-type mice recovered. RenTgMK mice exhibited more robust mucosal T_H_17 and T_H_1/T_H_17 responses and more profound colonic epithelial cell apoptosis compared to wild-type controls. Treatment with aliskiren (a renin inhibitor), but not hydralazine (a smooth muscle relaxant), ameliorated colitis in RenTgMK mice, although both drugs normalized blood pressure. Chronic infusion of angiotensin II into wild-type mice mimicked the severe colitic phenotype of RenTgMK mice, and treatment with losartan [an angiotensin type 1 receptor blocker (ARB)] ameliorated colitis in wild-type mice, confirming a colitogenic role for the endogenous RAS. In human biopsies, pro-inflammatory cytokines were suppressed in patients with inflammatory bowel disease who were on ARB therapy compared to patients not receiving ARB therapy. These observations demonstrate that activation of the RAS promotes colitis in a blood pressure independent manner. Angiotensin II appears to drive colonic mucosal inflammation by promoting intestinal epithelial cell apoptosis and mucosal T_H_17 responses in colitis development.

Inflammatory bowel diseases (IBD), including ulcerative colitis (UC) and Crohn’s disease (CD), are complex disorders. Although their exact etiologies remain incompletely understood, it is believed that these disorders are caused and/or perpetuated by inappropriate and ongoing activation of the mucosal immune system driven by the presence of commensal bacteria. This aberrant response is facilitated by defects in mucosal barrier functions of the intestine and/or derangements in the mucosal immune system^1^. An impaired mucosal barrier that results from abnormal apoptosis of intestinal epithelial cells (IEC) or dysfunctional paracellular junctions can lead to inappropriate invasion of luminal bacteria and antigens into the lamina propria, triggering aberrant mucosal immune responses^2^,^3^. Increased IEC apoptosis has been reported in patients with UC and CD^4^,^5^,^6^ as well as in murine models of colitis^7^,^8^. At the molecular level, p53 and p53-upregulated modulator of apoptosis (PUMA) are known to promote IEC apoptosis^9^,^10^.

The mucosal innate immune system, including dendritic cells, macrophages, neutrophils and innate lymphoid cells, constitutes an interface for monitoring the luminal environment and relaying signals to initiate adaptive immune responses. Thus, excessive activation of adaptive immunity is believed to be the more proximate driver of colitis^11^. It is known that CD4^+^ T_H_1, T_H_17 and T_H_2 T cell lineages all play critical roles in mucosal inflammation. T_H_1 cells produce interferon (INF)-γ and interleukin (IL)-12p40 and are thought to be predominantly involved in CD, T_H_2 cells secret IL-4, IL-5 and IL-13 and are more involved in UC, and T_H_17 cells produce IL-17, IL-21, IL-22, IL-6 and tumor necrosis factor (TNF)-α and are involved in both CD and UC^12^. T_reg_ cells, which produce anti-inflammatory cytokines such as IL-10, counter the effects of T helper cells. Foxp3^+^ T_reg_ cells and RORγt^+^ T_H_17 cells are derived from common CD4^+^ naïve precursor cells in the mucosa, which are driven by intestinal dendritic cells^13^,^14^. The balance between T_reg_ and T_H_17 cells is important for maintaining mucosal homeostasis^15^. T_H_17 cells are highly plastic and can further differentiate to T_H_1/T_H_17 cells that co-produce INF-γ and IL-17^16^.

The renin-angiotensin system (RAS) plays a central role in the regulation of vascular tone and salt and fluid balance. The RAS is a multi-component cascade with renin as the rate-limiting enzyme that cleaves angiotensinogen to angiotensin (Ang) I, which is further cleaved by angiotensin-converting enzyme (ACE) to Ang II. The later is the main effector of the RAS that acts by binding to angiotensin receptors (AT1 and AT2) found in many cell types including intestinal epithelial cells and mucosal immune cells^17^,^18^. AT1 receptor is also expressed in intestinal stromal fibroblasts^19^. Derangements in the RAS are known to be involved in numerous renal and cardiovascular diseases, but its role in gastrointestinal disorders such as IBD is less unclear. Circumstantial evidence suggests an involvement of the RAS in the pathogenesis of colitis. For example, increased colonic mucosal Ang I and II concentrations were reported in CD patients with active inflammation^20^. Genetically mutant mice with deletion in the AT1 receptor or the angiotensinogen gene developed less severe colitis than wild-type mice in experimental colitis models^21^,^22^,^23^. However, studies on the role of the RAS in colitis remain scarce in the literature and the underlying mechanism connecting the RAS and colitis is unknown. Here we use a transgenic mouse model that overproduces active renin to explore the effect of RAS activation on the development of colitis. Our study demonstrates that the renin-Ang II cascade promotes colitis by stimulating apoptosis of IECs and mucosal T_H_17 responses, and this effect is independent of blood pressure changes.

## Results

### Renin overexpression dramatically increases susceptibility to colitis

**Figure 1:**
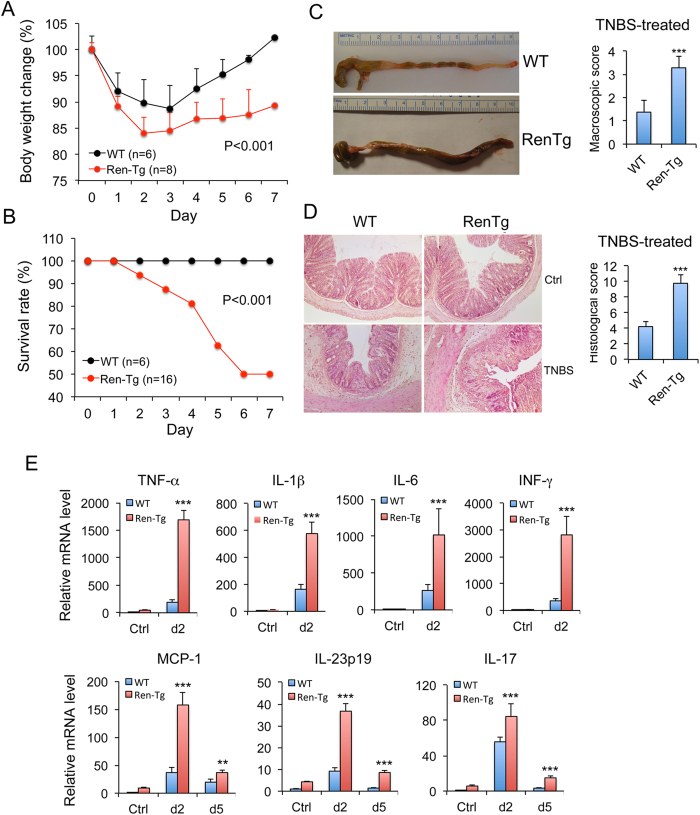
Renin overexpression promotes colitis in TNBS-induced colitis model. (**A**) Changes of body weight (% of original body weight) over time (day) in WT and RenTg mice following TNBS treatment. P < 0.001 by log-rank test; n = 6–8. (**B**) Survival curves of WT and RenTg mice following TNBS treatment; P < 0.001 by log-rank test; n = 6–16. (**C**) Gross morphology and macroscopic damage score of the large intestines from WT and RenTg mice on day 6 after TNBS treatment. (**D**) H&E staining of colon sections and colonic histological damage score for WT and RenTg mice on day 6 after TNBS treatment. Original magnification 100x. ***P < 0.001 vs. WT; n = 8–9 in each genotype. (**E**) Mucosal pro-inflammatory cytokine production quantified by qPCR in untreated WT and RenTg controls and on days 2 and 5 (d2 and d5) after TNBS treatment. **P < 0.01; ***P < 0.001 vs. WT.

RenTgMK (RenTg) mice produce and secret active renin from the liver, which is not regulated by the body’s salt and fluid status. Therefore, the transgenic mice have a high level of circulating Ang II and develop hypertension^24^, but grow normally and do not show any obvious symptoms. We used the RenTg mice as a model to explore the effect of chronic RAS activation on colitis development. Following intrarectal 2,4,6-trinitrobenzene sulfonic acid (TNBS) instillation both RenTg and wild-type (WT) littermates developed gradual weight loss, but the weight loss was more dramatic in RenTg mice compared with WT littermates. Whereas WT mice recovered their body weight within the following 7 days, RenTg mice failed to do so (Fig. 1A). In fact, about 50% RenTg mice died within these 7 days, but none of WT mice died (Fig. 1B). Compared with WT mice, RenTg mice showed more severe diarrhea and rectal bleeding, and their colon appeared markedly swollen and the colonic wall thickened with no visible fecal pellet formation, and RenTg colons had much higher macroscopic damage score than WT colons (Fig. 1C). Histological examinations confirmed more severe focal ulceration and impaired crypt regeneration in the distal colon of TNBS-treated RenTg mice compared to WT controls, along with thickened colonic walls, and TNBS-treated RenTg colons had significantly higher histological scores on day 6 (Fig. 1D). As expected, much higher levels of mucosal pro-inflammatory cytokines and chemokines (TNF-α, IL-1β, IL-6, INF-γ, MCP-1, IL-23p19 and IL-17) were produced in the RenTg colons compared to WT controls (Fig. 1E), consistent with the higher histological and clinical inflammation scores seen in the RenTg colons. We also measured intestinal mucosal permeability in TNBS-treated mice. When the mice were rectally administrated with FITC-dextran on day 2 before histological damage was detectable, RenTg mice had higher serum FITC-dextran concentrations than WT controls (Supplementary Fig. 1), suggesting a more leaky mucosal barrier in RenTg mice in the early stages of colitis development.

### RenTg mice show robust TH17 response and intestinal epithelial apoptosis

**Figure 2:**
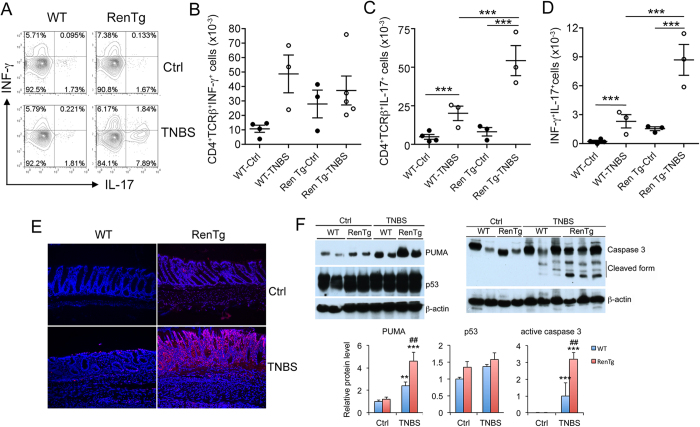
Renin overexpression promotes T_H_17 response and gut epithelial cell apoptosis in colitis model. (**A–D**) Lamina propria cells isolated from WT and RenTg mice on day 2 after TNBS treatment were analyzed by flow cytometry. Control mice received ethanol only. (**A**) Representative FACS plots gated on CD4^+^TCRβ^+^ cells. (**B**) Quantitation of INF-γ^+^ T cells; (**C**) Quantitation of IL-17^+^ T cells; (**D**) Quantitation of INF-γ^+^IL-17^+^ T cells. ***P < 0.001; n = 3–4 in each group. (**E**) Representative TUNEL staining of WT and RenTg distal colon sections on day 4 after TNBS treatment. Original magnification 100x. (**F**) Western blot analyses and densitometric quantitation of colonic mucosal PUMA, p53 and activated caspase 3 proteins in untreated and TNBS-treated WT and RenTg mice on day 2. **P < 0.01, ***P < 0.001 vs. Ctrl; ^##^P < 0.05 vs. corresponding TNBS-treated colons. All gels were run under the same experimental condition, and gel images are cropped for concise presentation. All uncut gel images are provided in Supplementary Information.

To further characterize the immune abnormalities accompanying the colonic inflammation in these mice we analyzed lamina propria cells by fluorescence-activated cell sorting (FACS) (Fig. 2A–D). Compared with WT mice, the number of INF-γ-producing CD4^+^ T cells was not significantly different in untreated RenTg mice (Fig. 2B), but RenTg mice treated with TNBS showed much more robust T_H_17 and T_H_1/T_H_17 responses. There was a more dramatic increase in IL-17-producing CD4^+^ T cells (Fig. 2C) and in CD4^+^ T cells that express both INF-γ and IL-17 (Fig. 2D) in RenTg colons on day 2 after TNBS treatment. In contrast, there was no significant difference in mucosal FoxP3^+^ T_reg_ cells (Supplementary Fig. 2A), IL-10 producing CD4^+^ T cells (Supplementary Fig. 2B) or IL-10-producing FoxP3^+^ T_reg_ cells (Supplementary Fig. 2C,D) between WT and RenTg mice. The highly dramatic increase in mucosal T_H_17 and T_H_1/T_H_17 responses, which are essential for colitis development^25^, is consistent with the severe colitis observed in RenTg mice.

As dysfunction of the mucosal epithelial barrier is a major mechanism leading to mucosal inflammation, we examined the apoptotic status of the mucosal epithelial barrier. TUNEL staining detected epithelial cell apoptosis in both WT and RenTg mice following TNBS treatment, but the extent and magnitude of apoptosis was much greater in RenTg mice (Fig. 2E). At the molecular level, the induction of PUMA, a major mediator for intestinal epithelial cell apoptosis^9^, and caspase 3 activation were both much more robust in TNBS-treated RenTg mice compared to WT counterparts (Fig. 2F). However, there was no significant difference in p53 levels in RenTg and WT mice. Excess epithelial apoptosis is expected to greatly impair the mucosal barrier, which likely accounts for the severe mucosal inflammation in RenTg mice.

We then used a cell culture system to assess whether Ang II promotes intestinal epithelial cell apoptosis. As reported previously^26^, TNF-α induced PUMA expression in HCT116 cells, a human colonic cancer cell line. Interestingly, Ang II could also induce PUMA expression in these cells, and a combination of TNF-α and Ang II had even more robust effects on the induction of PUMA (Supplementary Fig. 3A,B). These results suggest that Ang II has pro-apoptotic effects on colonic epithelial cells that can synergize with pro-inflammatory cytokines such as TNF-α that is increased in IBD.

### Renin promotes colitis independent of effects on blood pressure

**Figure 3:**
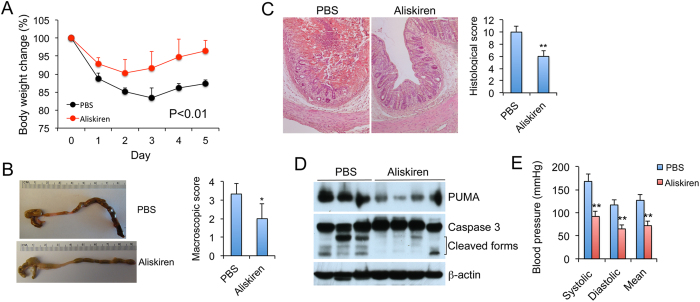
Renin inhibition ameliorates the severe colitis in RenTg mice. RenTg mice were pre-treated with PBS or aliskiren (10 mg/kg, i.p. daily) for one week before TNBS instillation. Aliskiren or PBS treatment continued after TNBS treatment. (**A**) Body weight changes (% of original body weight) over time (days) following TNBS treatment. P < 0.01 by log-rank test; n = 6 each group. (**B**) Gross morphology and colonic macroscopic damage score of the large intestines on day 5. (**C**) H&E staining of colon sections and histological damage score on day 5. Original magnification 100x. *P < 0.05; **P < 0.01 vs. PBS; n = 6 in each group. (**D**) Western blot analyses of mucosal PUMA protein and caspase 3 activation on day 3. (**E**) Blood pressure changes following aliskiren treatment. **P < 0.01 vs. corresponding PBS. All gels were run under the same experimental condition, and gel images are cropped for concise presentation.

**Figure 4:**
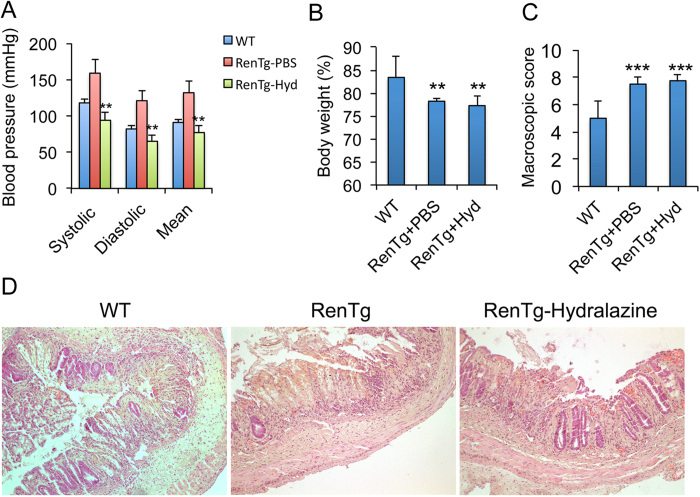
Renin promotes colitis independently of blood pressure. RenTg mice were pre-treated with PBS or hydralazine (10 mg/kg, i.p. daily) for one week before TNBS instillation. The treatment continued after TNBS treatment. WT mice serve as control. (**A**) Blood pressure changes following hydralazine treatment. **P < 0.01 vs. RenTg-PBS. n = 5–6 in each group. (**B**) Body weight changes (% of original body weight) on day 3; (**C**) Colonic macroscopic damage score; **P < 0.01; ***P < 0.001 vs. WT. n = 5–6 each group. (**D**) H&E staining of colon sections on day 5. Original magnification 100x.

To address whether the severe colitic phenotype of RenTg mice is caused by renin overproduction or by high blood pressure, we treated RenTg mice with renin inhibitor aliskiren or hydralazine, a vasodilator. As shown in Fig. 3, aliskiren treatment substantially accelerated body weight recovery following TNBS instillation (Fig. 3A), and attenuated morphological, macroscopic and histological damages of the colons (Fig. 3B,C). Mucosal pro-inflammatory cytokine production was suppressed. Aliskiren treatment also markedly reduced mucosal PUMA induction and caspase 3 activation (Fig. 3D). As expected, aliskiren treatment normalized the blood pressure of RenTg mice (Fig. 3E). In contrast, although hydralazine also normalized the blood pressure of Ren Tg mice (Fig. 4A), it had little effects on weight recovery (Fig. 4B) or macroscopic and histological injury caused by TNBS treatment (Fig. 4C,D). Taken together, these results indicate that renin activation promotes colitis by a mechanism independent of blood pressure changes.

### Chronic infusion of Ang II promotes colitis

**Figure 5:**
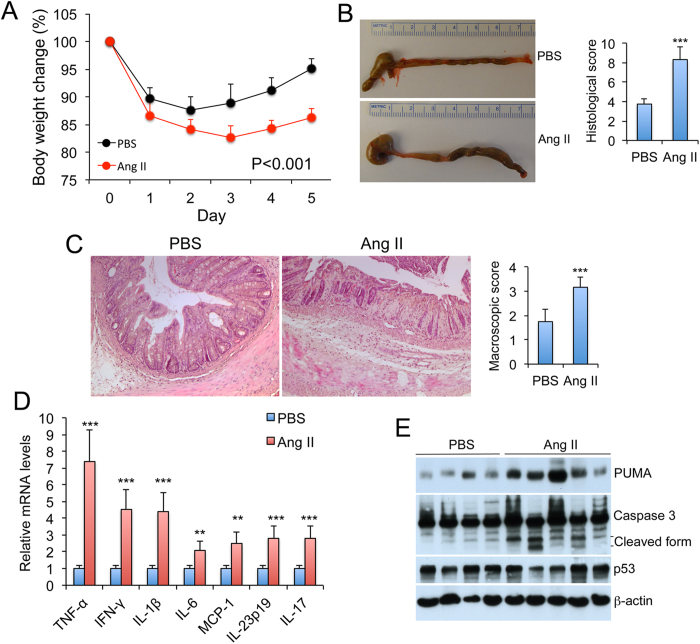
Chronic Ang II infusion promotes colitis. WT mice were infused with PBS or Ang II by osmotic minipumps for 15 days. TNBS was given on day 7 after infusion. (**A**) Body weight changes (% of original body weight) over time (days) following TNBS treatment. P < 0.01 by log-rank test; n = 6. (**B**) Gross morphology and macroscopic damage score of the large intestine on day 6. (**C**) H&E staining of colon sections and histological score on day 6. Original magnification 100x. ***P < 0.001 vs. PBS; n = 6 in each group. (**D**) Mucosal pro-inflammatory cytokine production quantified by qPCR on days 3 after TNBS treatment. **P < 0.01; ***P < 0.001 vs. PBS. (**E**) Western blot analyses of mucosal PUMA and p53 proteins and caspase 3 activation on day 3. All gels were run under the same experimental condition, and gel images are cropped for concise presentation.

To confirm that activation of the RAS promotes colitis, we directly assessed the effect of chronic Ang II infusion on colitis development in wild-type mice. Indeed, unlike PBS-infusion controls, WT mice receiving Ang II infusion failed to recover weight loss after TNBS treatment (Fig. 5A), and their colons showed more severe damage at the gross macroscopic and microscopic levels compared with PBS-infused counterparts (Fig. 5B,C). Mucosal pro-inflammatory cytokine levels were markedly increased in Ang II-treated mice (Fig. 5D), and PUMA induction and caspase 3 activation were also more robust (Fig. 5E).

### Blockade of AT1 receptor signaling ameliorates colitis

**Figure 6:**
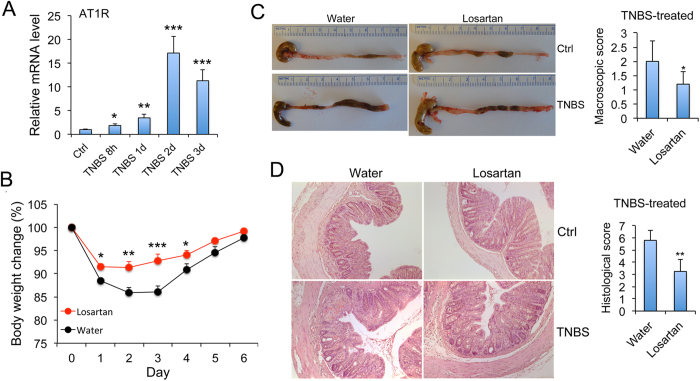
Blockade of AT1 receptor signaling ameliorates colitis in WT mice. (**A**) Time course expression of mucosal AT1 receptor in TNBS-induced experimental colitis. *P < 0.05, **P < 0.01; ***P < 0.001 vs. control. (**B**) Body weight changes (% of original body weight) over time (days). WT mice were fed regular water or losartan-containing (10 mg/ml) water for 7 days before TNBS instillation. Losartan treatment continued following TNBS treatment for 6 days before mouse sacrifice. *P < 0.05, **P < 0.01; ***P < 0.001 vs. water, n = 6 each group. (**C**) Gross morphology and macroscopic damage score of the large intestines on day 6. (**D**) H&E staining of colon sections and histological score on day 6. Original magnification 100x. *P < 0.05; **P < 0.01 vs. Water. n = 5–6 in each group.

Consistent with a critical role for the RAS in colitis pathogenesis, we observed that in the TNBS model with WT mice there was a dramatic induction of the AT1 receptor (>10 fold) in the colonic mucosa 2–3 days following TNBS treatment (Fig. 6A), suggesting an induction of the endogenous RAS. To explore further the effect of the endogenous RAS activation on colitis development, we used losartan, an AT1 receptor blocker, to block Ang II-AT1 receptor signaling in WT mice. As shown in Fig. 6, losartan-treated mice recovered their body weight much faster than untreated mice following TNBS instillation (Fig. 6B), and developed less severe colonic inflammation and mucosal ulcerations as judged by organ appearance and histological examination (Fig. 6C,D). Semi-quantitative macroscopic and histological scores of the colons confirmed that the injury was less severe in the losartan-treated colons (Fig. 6C,D). Real time PCR quantitation showed that the induction of pro-inflammatory cytokines and chemokines was significantly attenuated. Taken together, these studies indicate that activation of the endogenous RAS contributes to the development of colitis.

### IBD patients on ARB show reduced mucosal inflammation

**Figure 7:**
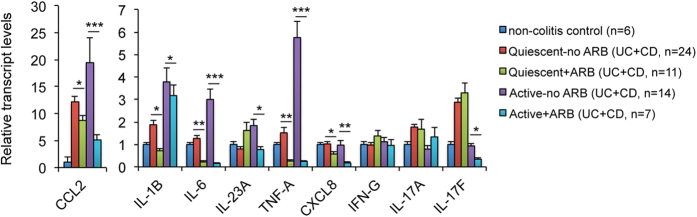
ARB therapy in IBD patients is associated with reduced mucosal inflammation. Real time PCR quantitation of pro-inflammatory cytokines and chemokines in mucosal biopsies from quiescent and active IBD patients (UC and CD) with or without ARB therapy. *P < 0.05; **P < 0.01; ***P < 0.001.

Finally, to address whether the results from these animal studies can be translated to human IBD, we compared pro-inflammatory cytokine levels in colonic biopsies between IBD patients on ARB and those who were not on ARB. RNAs from 11 quiescent and 7 active UC and CD patients who were on ARB were matched with 24 quiescent and 14 active UC and CD patients who were not on ARB. Because of the limited number of samples, transcript expression data from UC and CD patients were analyzed together. However, similar results were seen when UC and CD were analyzed separately. As shown in Fig. 7, real time RT-PCR quantitation confirmed that pro-inflammatory cytokines and chemokines (CCL2, IL-1β, IL-6, IL-23, TNF-α, IL-17A and IL-17F) were elevated in quiescent and active colitis biopsies. Most of these cytokines and chemokines (CCL2, IL-1β, IL-6, IL-23, TNF-α, IL-17F) were reduced in IBD patients on ARB therapy, especially for the active patients (Fig. 7). These data are largely in agreement with the notion that activation of the RAS is associated with colonic inflammation in human IBD patients, similar to our findings in renin transgenic mice or Ang II treated mice with colitis.

## Discussion

In this study we report that transgenic mice that over produce active renin from the liver are hyper susceptible to experimental colitis. This hyper susceptibility is dependent on active renin and independent of high blood pressure. We demonstrate that Ang II infusion worsens colitis, whereas blockade of the endogenous RAS ameliorates colitis. We also observe anti-inflammatory effects of ARB therapy in patients with IBD. Taken together, these data provide evidence strongly suggesting that the RAS is a colitogenic factor and RAS activation promotes colitis.

Probably because the gastrointestinal tract is not considered as a classic RAS target, our insights of the relationship between the RAS and IBD are very limited, and a literature search uncovered only a few relevant publications. An early study reported that colonic mucosal Ang I and II concentrations are increased in active CD biopsies, but the sample size was small (n = 20)^20^. Inconsistently, another study showed that serum ACE concentration was reduced in CD and UC patient population^27^. There are a few reports that show blockade of the RAS with an ACE inhibitor ameliorates colonic damage or colonic fibrosis in experimental colitis models^28^,^29^, and genetic deletion of AT1 receptor or angiotensinogen gene reduces the severity of colitis in mice^21^,^22^,^23^. Although these studies suggest a role for the RAS in colitogenesis, the underlying mechanism is unclear.

Here we used a renin transgenic mouse model to directly address whether RAS activation affects colitis development. The RenTg mouse line is a genetic equivalent of chronic Ang II infusion by a minipump that is a lifelong and noninvasive^24^. So it is an ideal model to address the question. Our studies showed that in TNBS colitis model the transgenic mice developed much more severe colitis compared with the wild-type control mice. The severe mucosal inflammation and colonic damage in the RenTg mice failed to recover during the course, leading to high mortality. We also showed that there was a dramatic induction of T_H_17 and T_H_1/T_H_17 immune responses in the early phase of colitis development in the transgenic mice, with much greater accumulation of IL-17-producing and INF-γ- and IL-17-producing CD4^+^ T cells in the lamina propria. Consistently, the transgenic mice displayed a greater induction of mucosal pro-inflammatory cytokines and chemokines in the early phase of colitis. T_reg_ cells, on the other hand, were not affected by the renin transgene. Thus, this shift in immune balance favors T_H_17 over T_reg_ response under RAS activation leading to robust mucosal inflammation in the RenTg mice. In agreement with the transgenic data, we also demonstrated that short-term infusion of Ang II in wild-type mice basically phenocopies the RenTg mice in terms of colitogenesis. Together these observations provide compelling evidence that RAS activation drives mucosal inflammation and promotes colitis.

How does the renin-Ang II cascade drive colonic inflammation? One possibility is that Ang II directly stimulates T_H_1/T_H_17 immune responses. In fact, it was previously reported that activated CD4^+^ T cells express high levels of the AT1 receptor and the RAS promotes T_H_1/T_H_17-mediated autoimmunity^30^. Another potential mechanism is that Ang II promotes intestinal epithelial cell apoptosis, which through colonic dendritic cells induces T_H_17 and T_H_1/T_H_17 cell differentiation. An elegant study by Blander’s group has demonstrated that bacteria- or LPS-induced apoptotic cells promote T_H_17 cell differentiation by TGF-β and IL-6^31^. During colitis development, T_H_17 cells can further differentiate to T_H_1/T_H_17 cells that produce both INF-γ and IL-17^25^. Indeed, massive epithelial cell apoptosis occurs in the transgenic mice after TNBS induction, and our *in vitro* cell culture data support that Ang II can stimulate PUMA-mediated pro-apoptotic pathway in epithelial cells, which is enhanced in the presence of TNF-α. PUMA has been well established as a key inducer of intestinal epithelial cell apoptosis^9^,^10^. Of course, these two potential mechanisms are not mutually exclusive. It is conceivable that both mechanisms are involved in colitogenesis driven by Ang II. Finally, the possibility that Ang II may alter colonic microbiome to influence colitis cannot be excluded. For example, Ang II could influence the microbiome profiles by regulating anti-microbial peptides, known to have a critical impact on intestinal mucosal inflammation^32^. Given the potential complex effects of the RAS on the gut, more studies are needed in the future to further elucidate the mechanisms whereby the RAS promotes the development of colitis.

Activation of the RAS increases blood pressure in the RenTg mice as expected^24^. Therefore, one important question is whether the severe colitic phenotype seen in the transgenic mice is secondary to high blood pressure. To address this question, we treated the transgenic mice with aliskiren or hydralazine. Aliskiren treatment, which inhibits renin enzymatic activity, markedly ameliorated mucosal inflammation. This proves that elevated renin activity is the cause of the severe colitis, but does not exclude the involvement of high blood pressure because aliskiren also lowers blood pressure. Importantly, when the blood pressure of the RenTg mice was normalized by hydralazine, which does not interfere with the RAS, colitis severity was not affected. These observations indicate that the stimulatory effect of the RAS on colitis is independent of blood pressure changes.

The RenTg mouse model is basically an “artificial” system that amplifies the effect of the RAS for investigation. Whether under normal conditions the endogenous RAS plays a role in colitis development needs to be addressed. We noticed that in wild-type mice mucosal AT1 receptor expression is dramatically increased in the early phase of colitis development. When wild-type mice were treated with losartan, which blocks AT1 receptor signaling, colonic inflammation was attenuated and colitis ameliorated. This is consistent with a few early studies that reported that an ACE inhibitor ameliorated colonic damage in experimental colitis models^28^,^29^. Therefore, the endogenous RAS does play a key role in the development of colitis. Intriguingly, as we reported previously, AT1 receptor is highly expressed in intestinal stromal fibroblasts^19^, and Ang II is known to stimulate myofibroblasts for fibrogensis through AT1 receptor^33^. As such, in addition to the gut epithelial cells and immune cells, the role of the intestinal fibroblasts in the development of colitis is worth further investigations.

One limitation of this study is that we have only studied the transgenic mice using the TNBS model, a T cell-driven colitis model resembling human CD^34^, but other experimental colitis models that are also commonly used in IBD research have not been tested for the RenTg mice. For example, the colitis model induced by dextran sulfate sodium (DSS) feeding, which disrupts the mucosal epithelial barrier, resembles UC in humans^35^. The colitis model induced by adoptive T cell transfer is believed to recapitulate the clinical and histopathological features of human CD better than the TNBS model^36^. Therefore, it needs to be cautious to generalize our conclusion with regards to the colitogenic effects of the RAS.

Inflammatory bowel diseases are devastating gastrointestinal disorders that are life long and without a cure at present. With increasing incidence of IBD worldwide, new and effective therapeutic strategies are needed for better management of IBD. The finding that the RAS promotes colitis is significant not only conceptually but also clinically, because the large number of drugs that target the RAS (some of which are very inexpensive) might potentially be used to treat IBD. In this regard, we showed that in IBD patients who were on ARB therapy generally exhibited lower pro-inflammatory cytokine expression in both quiescent and active states, and the inflammation score of their tissue biopsies was also trending lower. These data support the relevance of our animal observations. However, our human studies are only preliminary as the number of patients studied is small, and mucosal inflammation of these patients was not directly examined, which limit the significance of the conclusion. Another limitation is that, because of the small sample size, we combined the UC and CD samples in data analyses in order to obtain more meaningful results. There are clear differences in the etiology and manifestation of these two disorders. As such, the effect of the RAS on UC and CD could be different, and combining these two groups of samples could complicate the interpretation of the results. Therefore, future studies should be designed to validate our findings in human patients in both UC and CD groups. Prospective human intervention studies could be carried out to test the efficacy of anti-RAS drugs in the treatment of IBD.

## Methods

### Human biopsies

Human study subjects were recruited with written informed consent obtained from the participants or their guardians. The human studies and protocols were approved by the University of Chicago Institutional Review Board, in accordance with the Declaration of Helsinki of the World Health Organization, the Belmont Report and the Federal Policy for the Protection of Human Subjects. Patients with IBD (Crohn’s disease and ulcerative colitis) were recruited and consented at the time of endoscopy. Mucosal biopsies were obtained and placed in RNAlater (Qiagen) at the time of standard of care colonoscopy from areas with histological involvement of IBD. Total RNAs were extracted using the Allprep miRNA/RNA/DNA kit (Qiagen). Transcript levels of pro-inflammatory cytokines and chemokines were quantified by real time RT-PCR and compared between patients on ARB therapy to those not on ARB therapy after matching samples by disease type (CD or UC), disease status (quiescent or active), sex, age and race. We also included biopsies and RNA from normal healthy subjects as controls.

### Animal studies

All animal experimental protocols were approved by the Institutional Animal Care and Use Committee at The University of Chicago. All animal experiments were carried out in accordance with the Guide for the Care and Use of Laboratory Animals From National Research Council, Washington D.C. Transgenic mouse line RenTgMK (RenTg, on 129SV background) that carries a single copy of mouse renin transgene driven by liver-specific albumin promoter/enhancer^24^ was purchased from Jackson laboratory (Stock # 007853). Colitis was induced in RenTg mice and wild-type (WT) littermates using 2,4,6-trinitrobenzene sulfonic acid (TNBS) according to a protocol described previously^37^. Briefly, overnight fasted mice were treated under anesthesia with 100 mg/kg TNBS (Sigma) dissolved in 50% alcohol via intrarectal injection using a 1-ml syringe fitted with an 18-gauge stainless steel gavage needle, and control mice received 50% alcohol treatment. Body weights, stool consistence and rectal bleeding were monitored daily. Clinical scores and colonic damage scores were assessed as detailed previously^38^,^39^,^40^,^41^. Colons were collected immediately after sacrifice. The colons were fixed in 4% formaldehyde (pH 7.2) for histological analyses, or the mucosa was scraped to isolate RNAs or proteins. Colonic histological analyses were carried out using the “Swiss roll” method^42^ or using bread loafing cross sections, and histological scores were graded according to a previously published system^41^,^43^. Intestinal permeability was estimated by measuring FITC-dextran leak into the circulation as previously reported^44^,^45^. In this experiment, mice treated with TNBS for 2 days were fasted for 6 hours and then administered rectally with FITC-dextran (4000 Dalton) at 200 mg/kg. After 2 hours, blood was collected from the tail, and serum FITC-dextran content was determined using a microplate reader at 530 nm wavelength. In some experiments, RenTg mice and WT littermates were treated for two weeks with aliskiren or hydralazine (both by daily i.p. injection at 20 mg/kg/day, dissolved in PBS) before being treated with TNBS. Daily aliskiren or hydralazine injection continued after TNBS treatment. Mouse blood pressure was determined by the carotid artery cannulation method as described previously^46^.

To study the effect of angiotensin (Ang) II on colitis development, WT mice (on C57BL/6 background) were chronically infused with PBS or Ang II at 0.7 mg/kg/day for 14 days using ALZET osmotic minipumps. On day 7 after Ang II infusion, the mice were subjected to TNBS induction. To study the effect of AT1 blockade on colitis development, WT mice (on C57BL/6 background) were treated with losartan dissolved in drinking water at 10 mg/ml for 14 days. On day 7 after losartan feeding, the mice were subjected to TNBS induction.

### Flow cytometry

Lamina propria cells were isolated from the colon as described previously^47^. In brief, mice were killed, and the colons were dissected, cut open longitudinally and washed in cold PBS. The colons were cut into 1.5 cm pieces and washed in PBS containing 1 mM DTT for 10 min at room temperature on a shaker, followed by two washes with shaking in PBS containing 30 mM EDTA and 10 mM HEPES at 37 °C for 10 min. The tissues were then digested in RPMI 1640 medium (Invitrogen) containing DNase I (Sigma-Aldrich) (150 μg/ml) and collagenase VIII (Sigma-Aldrich) (150 U/ml) with 10% fetal bovine serum at 37 °C in a 5% CO_2_ incubator for 1.5 hrs. Digested cell suspensions were passed through a 70 μm cell strainer and separated by centrifugation on a discontinuous 40%/80% Percoll gradient at 2500 rpm for 20 min at room temperature. Cells were harvested for flow cytometry analyses. For cell surface staining, anti-CD16/32 antibody (eBioscience) was used to block non-specific binding to Fc receptors before staining. For intracellular staining, cells were fixed and permeablized using a Mouse Regulatory T Cell Staining Kit (eBioscience) according to the manufacturer’s protocol. For cytokine production, cells were stimulated with PMA (50 ng/ml) and ionomycin (500 ng/ml) for 4 hrs. Brefeldin A (2 μg/ml) was added 2 hrs before cells were harvested for analysis. Dead cells were excluded from the analysis using a Live and Dead Violet Viability Kit (Invitrogen). Anti-mouse CD3e FITC, anti-mouse CD4 Percp-Cy5.5, anti-Mouse/Rat IL-17A PE, anti-mouse/rat Foxp3 FITC, anti-Mouse/Human RORγt PE, anti-mouse IL-22 APC were purchased from eBioscience. Anti-mouse CD335 Percp-Cy5.5 (NKP46), anti-mouse IFN-γ Percp-Cy5.5, anti-mouse IL-10 PE, anti-mouse CD25 Pecy7, anti-mouse CD4 Pecy7 and anti-mouse TCR-β FITC were purchased from BD Pharmingen. Fluorescence-activated cell sorting (FACS) was performed in a FACSCanto II (BD Biosciences) and data analyzed by FlowJo software (Tree Star Inc).

### Histology

Freshly dissected colons were fixed overnight with 4% formaldehyde made in PBS (pH 7.2), processed and embedded in paraffin wax. Tissues were cut into 4-μm sections. Colonic morphology was examined by H&E staining as described^26^. To assess intestinal cell apoptosis, colon sections were subjected to TUNEL staining using an *In Situ* Cell Death Detection Kit (Roche Life Science) according to the manufacturer’s instruction.

### Cell culture

HCT116 cells were grown in DMEM supplemented with 10% fetal bovine serum. Cells were treated with TNF-α (100 ng/ml) or Ang II (100 ng/ml) alone or their combination, followed by isolation of protein lysates for Western blot analyses.

### RT-PCR

Total RNAs were extracted using TRIzol reagents (Invitrogen). First-strand cDNAs were synthesized using a ThermoscriptRT kit (Invitrogen). Conventional PCR was carried out in a BioRad DNA Engine (BioRad). Real time PCR was performed in a Roche 480 Real-Time PCR System, using SensiFAST SYBR No-Rox kits (Bioline). The relative amount of transcripts was calculated using the 2^−ΔΔCt^ formula^48^, normalized to GAPDH or actin transcript as an internal control. PCR primers for cytokines and chemokines and the RAS components were as reported previously^26^,^49^.

### Western blot

Proteins were separated by SDS-PAGE and electroblotted onto Immobilon-P membranes. Western blotting analyses were carried out as previously described^50^. The antibodies used in this study were against PUMA (Abcam), caspase 3 and p53 (Cell Signaling) and β-actin (Sigma).

### Statistical analysis

Data values were presented as means ± SD for mice and ± SEM for humans. The values were checked for normal distribution. For normally distributed data, statistical comparisons were carried out using unpaired two-tailed Student’s *t*-test for two group comparisons, and for three or more group comparisons two-way analysis of variance (ANOVA) was used with a Student-Newman-Keuls post-hoc test. Animal body weight changes and survival rate were analyzed by log-rank test. P < 0.05 were considered statistically significant.

## Additional Information

**How to cite this article**: Shi, Y. *et al.* Activation of the Renin-Angiotensin System Promotes Colitis Development. *Sci. Rep.* **6**, 27552; doi: 10.1038/srep27552 (2016).

## Supplementary Material

Supplementary Information
