# Supplementary material for: Activation of the Renin-Angiotensin System Promotes Colitis Development
Source: Sci Rep. 2016 Jun 8;6:27552. doi: 10.1038/srep27552 (PMC4897651; doi:10.1038/srep27552)
Supplement: Supplementary Information [file srep27552-s1.pdf]

Activation of the Renin-Angiotensin System Promotes Colitis Development

Yongyan Shi, Tianjing Liu, Lei He, Urszula Dougherty, Li Chen, Sarbani Adhikari, Lindsay Alpert, Guolin Zhou, Weicheng Liu, Jiaolong Wang, Dilip K. Deb, John Hart, Shu Q. Liu, John Kwon, Joel Pekow, David T. Rubin, Qun Zhao, Marc Bissonnette, and Yan Chun Li

Supplementary figure legends

Supplementary Figure 1. Mucosal permeability test. Blood FITC-dextran concentration in untreated and TBNS-treated WT and RenTg mice on day 2. ** $P<0.01$, ***$P<0.001$ vs. corresponding control; ## $P<0.01$ vs. WT; n=6 in each group.

Supplementary Figure 2. FACS analysis of T_{reg} cells. Lamina propria cells isolated from WT and RenTg mice on day 2 after TNBS treatment for FACS analysis. (A) Percentage of $Foxp3^{+}$ cells in $CD4^{+}CD25^{+}$ cells; (B) Percentage of IL-10 producing $CD4^{+}$ cells; (C and D) Quantitation of $Foxp3^{+}IL\text{-}10^{+}$ T cells. Flow cytometry plots were gated on $CD4^{+}CD25^{+}Foxp3^{+}$ cells (C) and percentage of $IL\text{-}10^{+}$ cells in $CD4^{+}CD25^{+}Foxp3^{+}$ cells (D).

Supplementary Figure 3. HCT116 cells were treated with TNF-α, Ang II or their combination, and PUMA expression was analyzed by Western blotting (A) and quantified by densitometry (B).

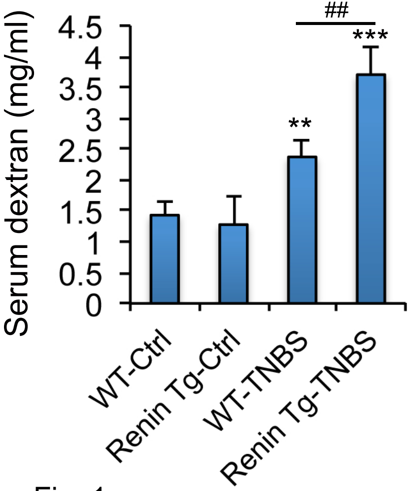
Serum dextran (mg/ml)
4.5
4
3.5
3
2.5
2
1.5
1
0.5
0
##
***
**
WT-Ctrl
Renin Tg-Ctrl
WT-TNBS
Renin Tg-TNBS

Sup. Fig. 1

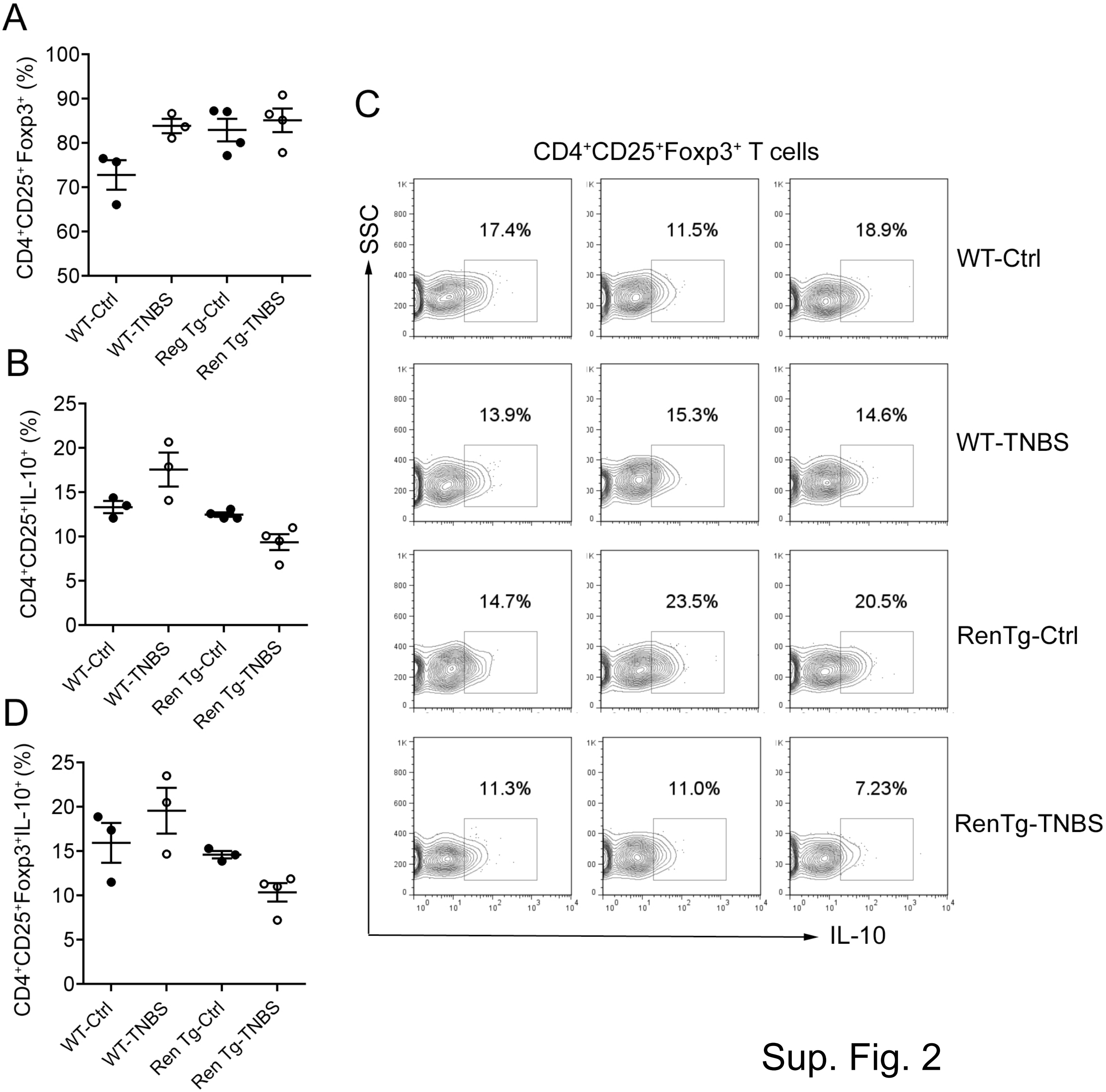

A
CD4+CD25+Foxp3+ (%)
100
90
80
70
60
50
WT-Ctrl
WT-TNBS
Reg Tg-Ctrl
Ren Tg-TNBS
B
CD4+CD25+IL-10+ (%)
25
20
15
10
5
0
WT-Ctrl
WT-TNBS
Ren Tg-Ctrl
Ren Tg-TNBS
C
CD4+CD25+Foxp3+ T cells
SSC
IL-10
17.4%
11.5%
18.9%
WT-Ctrl
13.9%
15.3%
14.6%
WT-TNBS
14.7%
23.5%
20.5%
RenTg-Ctrl
11.3%
11.0%
7.23%
RenTg-TNBS
D
CD4+CD25+Foxp3+IL-10+ (%)
25
20
15
10
5
0
WT-Ctrl
WT-TNBS
Ren Tg-Ctrl
Ren Tg-TNBS

Sup. Fig. 2

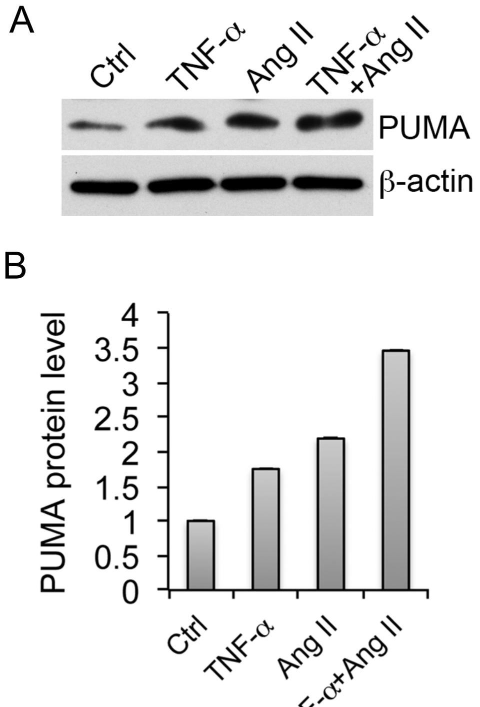
A
Ctrl
TNF-α
Ang II
TNF-α
+Ang II
PUMA
β-actin
B
PUMA protein level
4
3.5
3
2.5
2
1.5
1
0.5
0
Ctrl
TNF-α
Ang II
TNF-α+Ang II

Sup. Fig. 3

Activation of the Renin-Angiotensin System Promotes Colitis Development

Yongyan Shi, Tianjing Liu, Lei He, Urszula Dougherty, Li Chen, Sarbani Adhikari, Lindsay Alpert, Guolin Zhou, Weicheng Liu, Jiaolong Wang, Dilip K. Deb, John Hart, Shu Q. Liu, John Kwon, Joel Pekow, David T. Rubin, Qun Zhao, Marc Bissonnette, and Yan Chun Li

(Uncut gels)

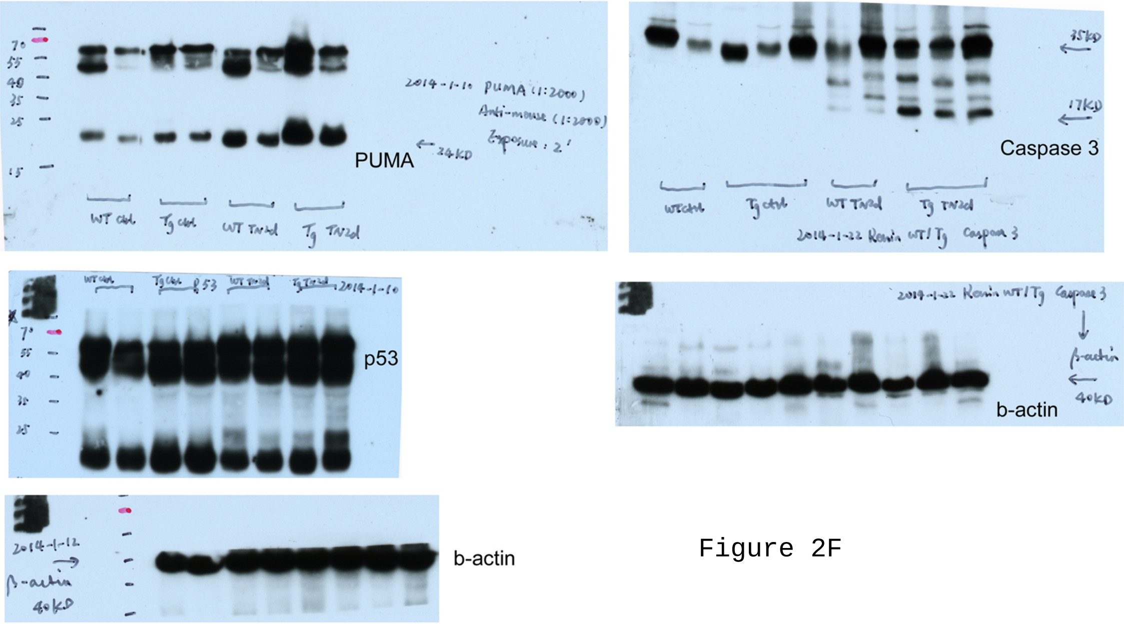
PUMA
2014-1-10 PUMA (1:2000)
Anti-mouse (1:2000)
Exposure: 2'
24KD
WT ctrl
Tg ctrl
WT TN2d
Tg TN2d
Caspase 3
35KD
17KD
2014-1-22 Kevin WT/Tg Caspase 3
p53
b-actin
40KD
2014-1-12
b-actin

Figure 2F

Figure 3D

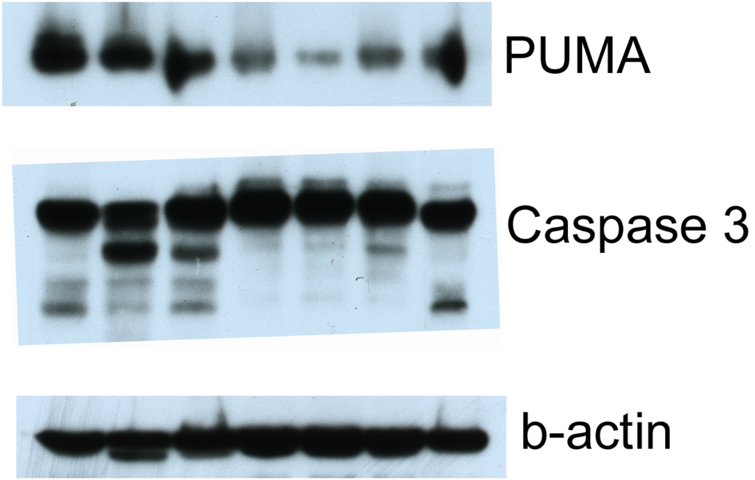

Figure 5E

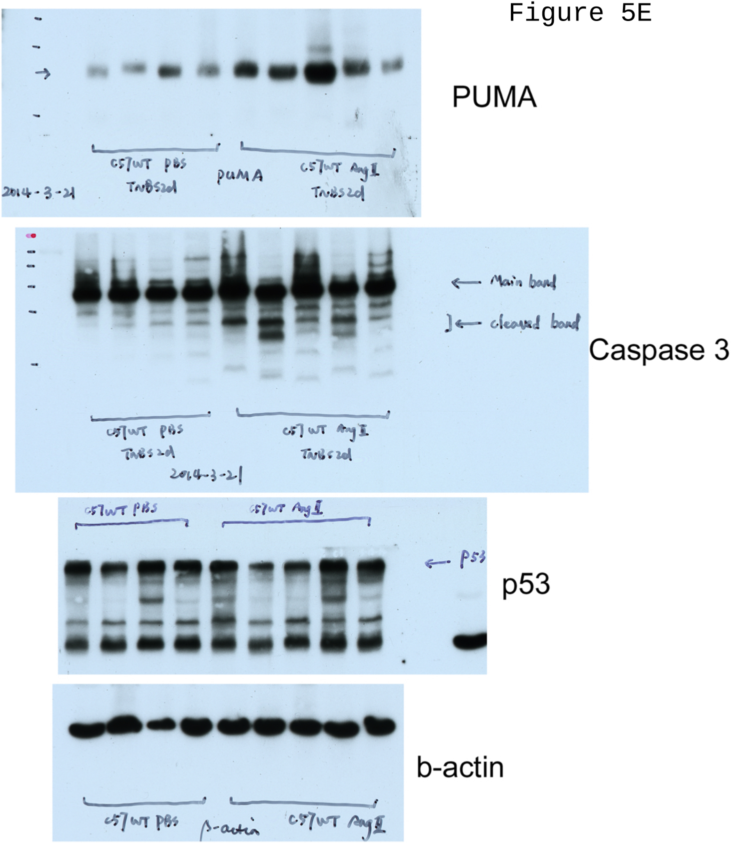
PUMA
C57WT PBS
TNBS 2d
PUMA
C57WT Ang II
TNBS 2d
2014-3-21
Caspase 3
Main band
cleaved band
C57WT PBS
TNBS 2d
C57WT Ang II
TNBS 2d
2014-3-21
p53
C57WT PBS
C57WT Ang II
p53
b-actin
C57WT PBS
β-actin
C57WT Ang II
